# Southern Dental Association

**Published:** 1884-08

**Authors:** 


### SOUTHERN DENTAL ASSOCIATION.

SIXTEENTH ANNUAL MEETING, HELD AT LEXINGTON, KY., MAY 6, 7, 8 AND 9, 1884.

REPORTED EXPRESSLY FOR THE INDEPENDENT PRACTITIONER.

(Continued from page 374.)

### AMALGAMS.

*The President*—Stated that Dr. Patrick had a letter from an officer of the Ordnance Department in reference to the swelling of amalgam in the mouth, instead of its contracting. He proposed it should be read.

*Dr. Patrick*—Stated that in a paper read in Boston, he had taken the ground that amalgams were poor things. In the course of his reading he never could get anything satisfactory as to the nature of amalgams. His experiments were so at variance with his authorities that they were very unsatisfactory. His paper, published by the American Dental Association, cost him a great deal of research. That paper, throughout all the journals, had never received a particle of attention, though he had been particular in sending it to every dental journal in America and Europe. It did not receive even a direct acknowledgment, except in one or two instances. That pamphlet was either full of error or full of truth. In either case it deserved attention; but the same men who ignored it will talk by the hour about dental education. It had been given him as a reason for this non-attention that the proprietors of most of our journals have too much amalgam to sell. He did not believe it, for he gave these men credit for business capacity, and believed the time would never come when there will be no amalgam used. It is a good democratic material to use, for it brings all dentists on a level. He had noticed that articles on the other side always received attention. He had been consulting a reliable authority on ship-building, railroad construction, etc.; it was the engineer's text-book " Haswell." In it he read that "all amalgams expand," and yet in every advertisement of an amalgam it is warranted to contract. He wanted to know the reason of expansion, and wrote to a friend at Washington, Lieutenant Kossuth Niles, of the Ordnance Department, whose answer was that he could not find anything on the expansion of amalgams in cooling and mixing, and that he had spoken to Dr. Woodbridge, whose experience was that amalgams expand in cooling and mixing.

In answer to a question as to whether that could not be accounted for in the change of the cells to globes, Dr. Patrick said there were so many different kinds of amalgams that it was difficult to tell. He could not answer without some particular amalgam was specified.

*Dr. Patrick*—Asserted that an easy flowing solder should never be mixed with any base metal. Jewelers or goldsmiths never use any base metal in their solder. Take 20-carat gold and use 18-carat solder. You cannot use solder that will free you from the responsibility of the blow-pipe. He read this formula: gold, 89; silver, 7; copper, 4. He advised that solder never be kept on hand. Prepare the plate first, then make the solder from that gold; but never keep it over. Melt the residue up with the plate, and make fresh solder for the next case.

In answer to a question he said, if 22-carat gold be used in the formula, that will make about 19-carat solder, and you cannot tell it from the gold plate when polished. In filagree-work requiring fine solder, the work is boiled in nitric acid to bring out the color.

*Prof. H. A. Smith*—(of Cincinnati), supposed amalgams must not be used, and spoke ironically against them.

*Dr. How*—Knew that if thin glass beads were filled with some amalgams they would be broken by the setting of the amalgam.

*Dr. Smith*—Supposed that was on account of the particles taking the spherical form, and spoke further of the utility of amalgams.

*Dr. Rehwinkel*—Spoke of crowning teeth when a root is broken under the gum. In filling with amalgam that comes to the edge of the gum, the latter is irritated, and one is never sure of the stability of the material at that point. In case the inner or outer cusp is broken, he takes a thin sheet of platinum to form a band.

Binding a thin copper band round the root, to reach under the gum, he fits it closely to the stump, fills the open end with plaster-of-Paris and when it has set withdraws it. This gives him a complete impression, and by filling with fused metal the exact model is obtained, over which the platinum band can be fitted. He cuts so that the crown will overlap, thus covering, protecting and strengthening the root and amalgam joint.

*The President*—Inveighed against the use of ill-smelling copper when platinum will do, and is so much cleaner.

*Dr. Rehwinkel*—Supposed copper was more economical, hence its use.

*The President*—Then called attention to the new German practice of filling teeth—the rotary method. Dr. Rehwinkel had translated an article from the German on the subject, and he would like to hear from him.

*Dr. Rehwinkel*—Stated that he had no practical experience of the method. Very dry amalgam could be used. He had not used the instruments for gold. It required a great deal of practice to become expert with the instruments and gain confidence. Langstaff had investigated the method and spoke highly of it, and Nicolet has adopted it and claims he can do anything with it. Experiments had been made in filling teeth out of the mouth, to find the adaptability of the filling. A matrix is indispensable, as a bud-shaped condenser is used.

*The President*—Said he had got a set of instruments, and had tried them on teeth out and in the mouth. There was much of good in the rotary method in applying gold to the side of a cavity. He had not been successful, but could not say whether the secret of success was in the peculiar gold used or the manner of using it.

*Dr. Rehwinkel*—Thought the gold used was something like our soft silver.

*Dr. How*—Stated that he had been engaged in some experiments but had not finished them. He was of the opinion that the instruments worked better after the gold had entered into their surface.

TRAFFIC IN DENTAL DIPLOMAS.

*Dr. F. H. Rehwinkel*—(of Chillicothe, Ohio), referred to the traffic in diplomas, carried on by the Wisconsin Dental College. He recently had a letter from Dr. Petermann, of Frankfort-on-the-Main, stating that this disgraceful business is in full blast again, complaining of the utter indifference of the dental profession, and that no effort is made to stop the dirty commerce. It would be remembered that in the case of the Buchanan College of Pennsylvania, it was left to a member of the press to begin the fight. Dr. Petermann engaged in a seven-years war, and he is now ready to take up the gauntlet with the Wisconsin Dental College, and wants the aid of the profession in America. These diplomas are not worth the paper on which they are written. Their issue may be legal, because of the lax laws of that State. Every state should take it up and bring pressure to bear on the State of Wisconsin to have the charter repealed. About two years ago an offer was made to him of a diploma on parchment for twelve dollars, and he felt disposed to resent it. He believed it to be the duty of the profession to knock the traffic on the head. As the Wisconsin College has a legal existence it would be necessary to move, as in the case of the Buchanan College, through the Legislature of the State. The government of Germany has already taken notice of it, and the matter will probably be made one of diplomatic correspondence, but it would be well to take action to strengthen the hands of the Germans.

*The President*—Hoped action would be taken, and called upon Professor Morgan, of Nashville, Tenn., who condemned the manner of issuing low-class diplomas. He did not know how to reach the evil, which is of deep interest to the profession in the eyes of Europe, unless an expression could be drawn forth from the State of Wisconsin. Everybody knew the dirtiness of the case, and that the practice degrades the profession in the United States.

It was, on motion, resolved, that a committee of three be appointed to draft resolutions in respect to the matter; and the President appointed Dr. A. O. Rawls, Professor W. H. Morgan, and Dr. B. H. Catching.

(TO BE CONTINUED.)

